# Lethal pneumatosis coli in a 12-month-old child caused by acute intestinal gas gangrene after prolonged artificial nutrition: a case report

**DOI:** 10.1186/1752-1947-2-238

**Published:** 2008-07-24

**Authors:** Stefan Kircher, Rupert Wössner, Hans-Konrad Müller-Hermelink, Hans-Ullrich Völker

**Affiliations:** 1 Institute of Pathology, University Würzburg, Josef-Schneider-Straße, D-97080 Würzburg, Germany; 2 Department of Paediatrics, University Würzburg, Germany

## Abstract

**Introduction:**

Pneumatosis coli is a rare disease with heterogeneous symptoms which can be detected in the course of various acute and chronic intestinal diseases in children, such as necrotizing enterocolitis, intestinal obstruction and intestinal bacteriological infections.

**Case presentation:**

We report the case of a 12-month-old boy who died of pneumatosis coli caused by an acute intestinal gas gangrene after prolonged artificial alimentation.

**Conclusion:**

While intestinal gas gangrene is a highly uncommon cause of pneumatosis coli, it is important to consider it as a differential diagnosis, especially in patients receiving a prolonged artificial food supply. These patients may develop intestinal gas gangrene due to a dysfunctional intestinal barrier.

## Introduction

Pneumatosis coli (PC) is a rare entity which was first described by DuVernoi in 1730 [1]. Clinically, PC is associated with multiple submucosal or subserosal gas-containing cysts in the wall of the intestinal tract. The aetiology of PC has been divided into primary (idiopathic) and secondary forms (resulting from other intestinal diseases). Important causes of PC in children are necrotizing enterocolitis [2], intestinal obstruction, for example, in pyloric stenosis, meconium ileus and Hirschsprung's disease; and ischaemia, for example, due to intussusception or volvulus, intolerance to carbohydrates or lactose, or steroid therapy [3]. A further cause of secondary PC is an intestinal bacteriological infection, especially with *Clostridium perfringens *or *C. septicum *[4], which can result in intestinal gas gangrene. Some cases of intestinal gas gangrene have been reported in the recent literature that have been found incidental to trauma, immunodeficiency such as malignancy, haematological disease and diabetes mellitus [5]. Yet another cause of secondary PC is an overload of *C. perfringens *resulting from ingestion of contaminated food – 'pigbel' disease [6].

To the best of our knowledge, no case of intestinal gas gangrene has been described following artificial nutrition. Here we present the case of a 12-month-old boy who died of intestinal gas gangrene after prolonged artificial alimentation.

## Case presentation

We report the case of a 12-month-old boy who suffered from perinatal asphyxia during delivery, resulting in severe hypoxic encephalopathy with tetraparesis and epilepsy. In addition he suffered from considerable dysphagia from birth. For this reason his parents provided artificial nutrition by a stomach tube at home. The supplied food consisted of hydrolysed milk formula based on amino acids and a natural thickening agent composed of carob seed flour. The patient had a history of abdominal pain, fever and nausea one week prior to being referred to our paediatric clinic with symptoms of acute abdomen, increasing fever (up to 42°C), cyanosis and epileptic seizures.

### Clinical course

The clinical diagnostic procedures demonstrated respiratory insufficiency with decreased arterial oxygen saturation (pO_2 _< 80%). The peripheral blood showed the following values: pH 7.23; leukocytes 28,200/μl; thrombocytes 84,000/μl; haemoglobin 14.9 g/dl; lactate 6.6 mmol/l; and increasing transaminases.

**Figure 1:**
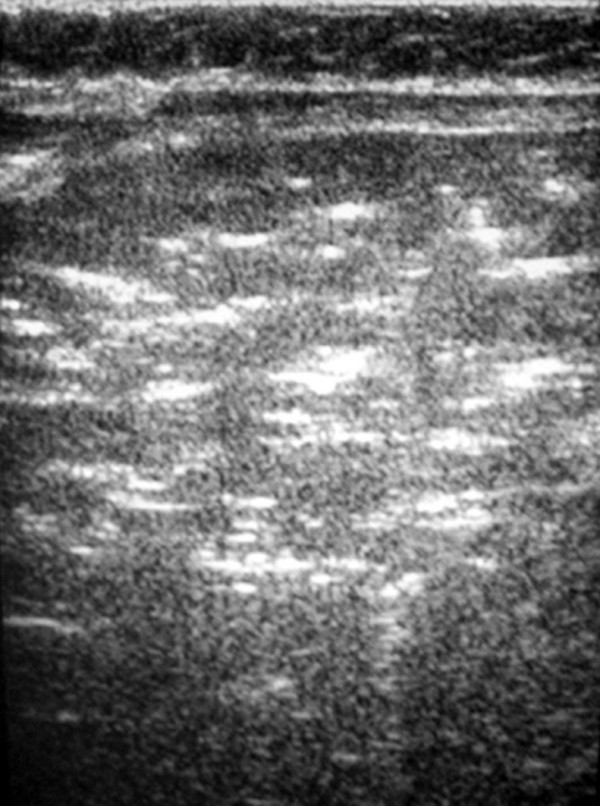
Ultrasonography showing free air bubbles in the hepatic blood vessels.

In the abdominal X-ray bloated bowel and pneumatosis coli were detected; ultrasonography showed free air bubbles in the hepatic blood vessels and the portal vein (Figure 1). Blood culture and cerebrospinal fluid were abacterial. *Pseudomonas aeruginosa *was found in the pharynx.

Due to clinical presentation of sepsis, the patient was intubated and transfused with NaCl, fresh frozen plasma and thrombocyte concentrate. Furthermore, antibiotic therapy was administered with cefotaxime, gentamicin, metronidazole and mezlocillin and catecholamines were prescribed due to insufficiency of the cardiovascular system. However, the patient showed a rapid deterioration and died after two attempts of resuscitation on the day of admission.

### Autopsy results

**Figure 2:**
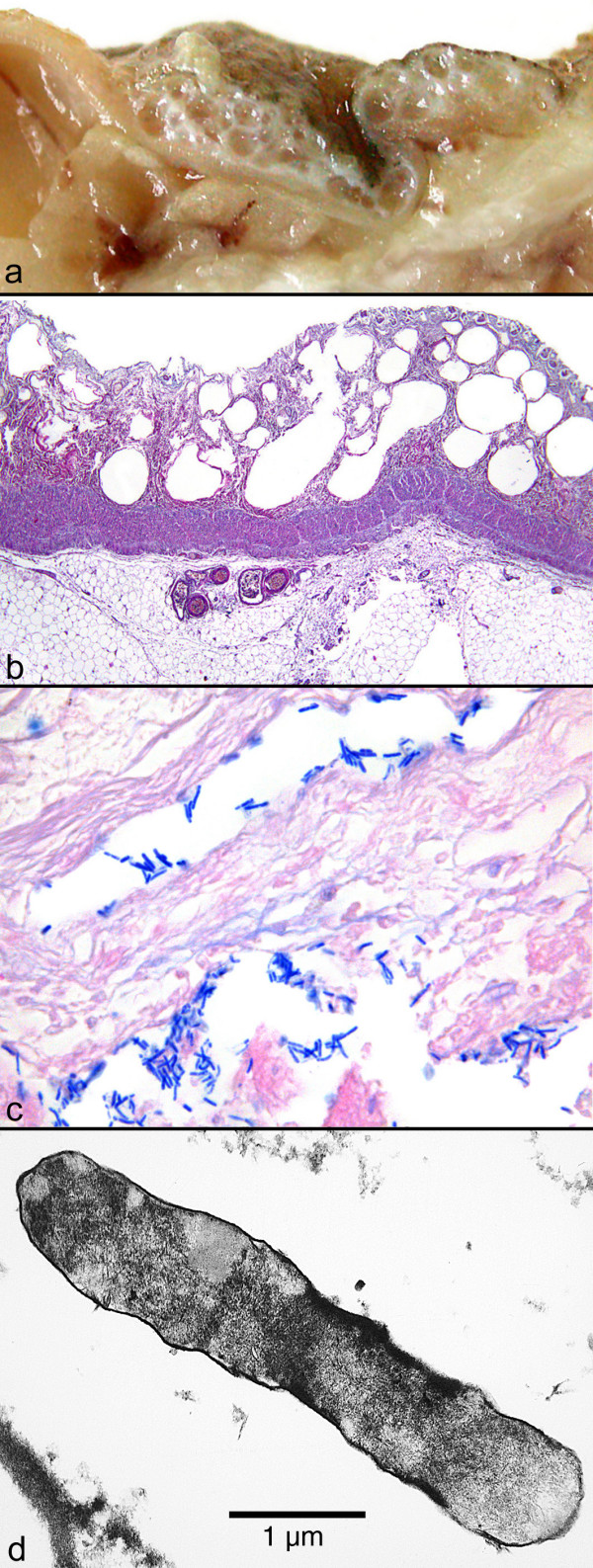
**Macroscopic, histological and ultrastructural assessment of small intestine tissue**. (a) Macroscopic picture of the oedematous intestinal wall with multiple submucosal and subserosal cysts. (b) Histological picture of the intestinal mucosa with areactive necrosis. (c) Gram stain of cysts with large rod-shaped bacteria. (d) Electron microscopic picture of a bacterium found in a submucosal cyst.

An autopsy was performed with permission from the parents. The examination revealed considerable obesity, with a size of 74 cm (25th percentile) and weight of 10.3 kg (65th percentile). After the abdomen was opened extensive subcutaneous and muscular oedema was found, but no ascites and no blood. The bowel was bloated and the small intestine in particular revealed an oedematous intestinal wall with multiple submucosal and subserosal cysts, corresponding to the typical macroscopic picture of a PC (Figure 2a). The gastric wall showed no pathological findings.

Histologically, the intestinal mucosa and submucosa showed marked areactive necrosis with no evidence of inflammatory infiltration, especially neutrophils (Figure 2b). Within and beneath the cysts, large rod-shaped bacteria were found. These were strongly positive in a subsequent Gram stain (Figure 2c). Scanning electron microscopy indicated that the bacteria were about 0.3 to 0.9 μm wide with blunt ends and without flagella (Figure 2d), consistent with *Clostridium *spp. There was no evidence of typical pseudomembranous colitis and no inflammatory infiltration in the necrotic mucosa consistent with the existence of *C. perfringens*. Molecular subtyping was not possible with the available material. These findings led to a conclusive diagnosis of intestinal gas gangrene.

Furthermore, acute haemorrhages were detected in the liver, kidneys and spleen, corresponding to a disseminated intravascular coagulation. The lungs showed slight focal signs of previous aspirations. Hepatic steatosis with hepatocellular fatty changes in 80% of hepatic tissue was detected. The other organs showed no pathologic changes. Consent was not given for a brain autopsy.

The cause of death was recorded as protracted haemodynamic shock following intestinal gas gangrene.

## Discussion

A possible cause of pneumatosis coli, apart from other predisposing diseases and conditions, is intestinal gas gangrene in the setting of an infection with *C. perfringens *or *C. septicum *[5]. We have presented the case of a 12-month-old boy who developed this disease after a prolonged supply of artificial nutrition. The nutrition applied by the boy's parents was hypercaloric with subsequent development of a severe infantile obesity and hepatic steatosis. To the best of the authors' knowledge, no regular medical or nursing controls were accepted. Intestinal gas gangrene is a rare disorder. To the best of our knowledge, no case has been reported previously as a complication of artificial nutrition.

*Clostridium *spp. are physiologically found in the gut as part of the normal flora, but usually they are unable to invade the intestinal wall. An altered permeability of intestinal barrier function is a precondition that may result in an infection with *Clostridium*, ultimately leading to intestinal gas gangrene. Possible causes for disorders in intestinal barrier function are inflammation, cytokines, hormones, toxins and hyperosmotic stress [7,8]. It is also recognized that artificial nutrition may result in significant alterations of epithelial barrier function [9], such as those observed in hogs fed with unpolished rice. It has been suggested that a relative deficiency of disaccharidase may prevent carbohydrate digestion resulting in increased bacterial fermentation and the development of PC [10]. In addition, hyperosmolar enteral or parenteral nutrition can lead to mucosal atrophy and impaired intestinal defence. Furthermore, a disruption of the normal bacterial flora may result [11] which could also improve the conditions for *Clostridium *spp.

## Conclusion

As PC is a disease with heterogeneous symptoms which can be detected in the course of many different acute and chronic intestinal diseases in children, such as necrotizing enterocolitis, intestinal obstruction and intestinal bacteriological infections, early recognition and management is important. With regards to differential diagnosis, even highly uncommon causes such as an intestinal gas gangrene should be considered. In this case the prolonged artificial nutrition may have played a major pathogenic role in the development of intestinal gas gangrene by impairing the intestinal barrier function, with consequent infection with *C. perfringens*.

## Abbreviations

PC: pneumatosis coli.

## Competing interests

The authors declare that they have no competing interests.

## Authors' contributions

SK drafted the manuscript. RW contributed the clinical findings. H–KM–H interpreted the pathological findings. SK and H–UV reviewed the manuscript. All authors read and approved the final manuscript.

## Consent

Written informed consent was obtained from the patient's next-of-kin for publication of this case report and accompanying images. A copy of the written consent is available for review by the Editor-in-Chief of this journal.
